# Sidetracked by trolleys: Why sacrificial moral dilemmas tell us little (or nothing) about utilitarian judgment

**DOI:** 10.1080/17470919.2015.1023400

**Published:** 2015-03-20

**Authors:** Guy Kahane

**Affiliations:** ^a^ Uehiro Centre for Practical Ethics, Faculty of Philosophy, Oxford University, Oxford, UK

**Keywords:** Morality, Moral decision-making, Sacrificial dilemmas, Utilitarian judgment

## Abstract

Research into moral decision-making has been dominated by sacrificial dilemmas where, in order to save several lives, it is necessary to sacrifice the life of another person. It is widely assumed that these dilemmas draw a sharp contrast between utilitarian and deontological approaches to morality, and thereby enable us to study the psychological and neural basis of utilitarian judgment. However, it has been previously shown that some sacrificial dilemmas fail to present a genuine contrast between utilitarian and deontological options. Here, I raise deeper problems for this research paradigm. Even when sacrificial dilemmas present a contrast between utilitarian and deontological options at a philosophical level, it is misleading to interpret the responses of ordinary folk in these terms. What is currently classified as “utilitarian judgment” does not in fact share essential features of a genuine utilitarian outlook, and is better explained in terms of commonsensical moral notions. When subjects deliberate about such dilemmas, they are not deciding between opposing utilitarian and deontological solutions, but engaging in a richer process of weighing opposing moral reasons. Sacrificial dilemmas therefore tell us little about utilitarian decision-making. An alternative approach to studying proto-utilitarian tendencies in everyday moral thinking is proposed.

## WHY TROLLEYS?

One of the welcome trends in recent social psychology and neuroscience has been the increasing interest in the processes and mechanisms that underlie moral cognition. Less obviously welcome is the dominant role given, within this research, to moral dilemmas where one must decide whether to sacrifice one person to save a greater number (for a review, see Christensen & Gomila, 2012). These sacrificial dilemmas were inspired by the thought experiments of moral philosophers involving runaway trolleys (Foot, 1967; Thomson, 1985), but in other variants they also include out of control epidemics, desperate survivors on a lifeboat, swinging cranes, and the like.

This research focus is rather puzzling. These hypothetical dilemmas are complex, far-fetched, and often convoluted. It would be strange to think that they offer the key to understanding moral judgment in general. If we wanted to identify the building blocks of moral judgment, it would presumably be more sensible to start by investigating simple instances of moral judgment such as the judgment that a malicious lie or bullying violence are wrong, and—giving special focus to developmental questions—work our way up from there. Eventually, we are likely to arrive at special cases where it can seem that lying and violence could nevertheless be permitted (whether because needed to prevent an even greater harm or for some other reason). Sacrificial dilemmas would thus be just a minor (if interesting) branch within a much broader inquiry, and their interpretation would depend on prior groundwork done on much simpler, more basic cases.^1^ ^1^For a battery of validated vignettes involving everyday moral situations, see Knutson et al. (2010).


So why this odd focus? One simple explanation is that one of the first neuroimaging studies of moral cognition (Greene, Sommerville, Nystrom, Darley, & Cohen, 2001) used these dilemmas and was published in a major journal, receiving a vast amount of attention. That attention led other researchers to employ this paradigm in other studies. And once a body of research grows around a paradigm, it is easier to build on it than to come up with a new experimental design. Soon everyone is using this paradigm, just because everyone else is. Needless to say, this sociological point is not a good reason to focus so much research on this peculiar paradigm.

A somewhat better reason for this research focus is that sacrificial dilemmas are widely seen as a way to shed light on the fundamental ethical division between utilitarian and non-utilitarian (or “deontological”) approaches to ethics—it is often assumed that by employing such dilemmas, we can uncover the psychological processes and neural mechanisms underlying these opposing ways of thinking about morality (Greene, Nystrom, Engell, Darley, & Cohen, 2004), and perhaps even resolve this fundamental ethical conflict (Greene, 2008; Singer, 2005). Engaging in such grand questions certainly seems more exciting than studying pedestrian moral judgments about everyday harm or dishonesty. However, the relation between sacrificial dilemmas and these philosophical debates is often misunderstood in this literature.

Researchers in this area often seem to assume that philosophers originally introduced “classical” sacrificial dilemmas in order to highlight the division between utilitarianism and deontology, and that such dilemmas play a key role in the dispute between these views.^2^ ^2^For example, Christensen and Gomila (2012) write that sacrificial dilemmas “were instrumental in arguing for the inconsistency of utilitarianism (or Consequentialism, in general) as an ethical theory” (p. 1251). However, the Foot and Thompson articles that first introduced these trolley cases are not at all concerned with this issue; they proceed on the assumption that utilitarianism is false. This however is a misunderstanding of the philosophical purpose of these thought experiments. The debate between utilitarians and their opponents has indeed often appealed to elaborate thought experiments and fanciful examples, both to criticize utilitarianism and to support it—thought experiments involving, for instance, archbishops and chambermaids in a burning building (Godwin, 1793/1926), the moral integrity of a chemist (Williams, 1973), a child drowning in a pond (Singer, 1972), or a rich uncle drowning in a bathtub (Rachels, 1975). But dilemmas involving runaway trolleys do not figure very prominently in this debate. They were first introduced, and most heavily discussed, as a problem *within* a strand of deontological ethics (Foot, 1967; Kamm, 2007; Thomson, 1985). To the extent that the aim of this recent empirical research on moral dilemmas is to use the hypothetical cases that most sharply divide utilitarians and their opponents, then this research may be focusing on the wrong examples.

It might be thought that sacrificial dilemmas nevertheless do present a contrast between a utilitarian view (sacrifice one to save a greater number) and opposing deontological view (it is wrong to do so), and as such can still shed light on this ethical division, even if their original philosophical purpose was somewhat different. I will argue however that it is a mistake to interpret the moral judgments of ordinary folk in terms of these philosophical theories. Ordinary responses to sacrificial dilemmas tell us little about utilitarianism or about any grand philosophical dispute.

## MORAL DILEMMAS, RIGHT OR WRONG

Some issues with the sacrificial dilemmas paradigm start at a basic level, and can already be traced to that first study, Greene et al. (2001). That study introduced a battery of “personal” and “impersonal” sacrificial dilemmas. Some of these dilemmas—such as the famous sidetrack and footbridge trolley cases—were directly based on philosophical thought experiments. But many were invented for the occasion, and, unfortunately, a significant proportion of these new dilemmas does not involve anything like a clear contrast between utilitarian and non-utilitarian options. For example, in one new “personal” dilemma subjects were asked whether it is morally appropriate to murder an annoying architect—an amoral action that neither utilitarianism nor its opponents would dream of sanctioning (Kahane & Shackel, 2008). That a battery including such scenarios would be associated with stronger activation in emotional parts of the brain is thus hardly a great discovery about deontological ethics (Kahane & Shackel, 2010).

This issue affects, to varying degrees, much of the original battery of personal dilemmas. Unfortunately, a great deal of subsequent research in this area—including some fairly recent studies—continues to use this problematic original battery of dilemmas to study moral judgment, wrongly classifying the judgment, for example, that it is appropriate to murder the annoying architect as a “utilitarian” judgment.^3^ ^3^The scope of the problem should not be overly exaggerated. Some studies have used only variants of the original trolley problems (see, e.g., Greene et al., 2008) or better controlled stimuli (e.g. Moore, Clark, & Kane, 2008).


This simple problem has not yet been sufficiently recognized, but some later research has more or less found a way around it. Koenigs, Kruepke, Zeier, and Newman (2012) introduced a distinction between “high” and “low” conflict personal dilemmas (that is, dilemmas on which there is significant disagreement between subjects and dilemmas on which there is near complete consensus), and Greene, Morelli, Lowenberg, Nystrom, and Cohen (2008) recommend focusing only on the former to study the contrast between “utilitarian” and “deontological” judgment. Since a large majority of subjects reject the deeply immoral option offered in some of the most problematic dilemmas (e.g. to murder the architect), there was a strong consensus on these dilemmas and they are classified as “low” conflict, and thus appropriately excluded by later studies that focus only on “high” conflict dilemmas.

However, while the focus on “high” conflict dilemmas is a step forward, it is also misleading, and it only partly addresses the problem. A flaw in the content of a set of dilemmas can only be fully addressed by reclassifying the dilemmas in *terms* of their content. Whether a dilemma involves a genuine contrast between utilitarian and deontological choices surely depends on the content of the dilemma, not on the degree of consensus about it (Kahane & Shackel, 2010). Opinions may be strongly divided about a moral dilemma even if it doesn’t involve a sharp contrast between utilitarian and deontological options (see below for some examples) while there may be strong consensus against (or for that matter, for) the utilitarian option in dilemmas that do involve a genuine contrast between utilitarian and deontological views. In fact, such a strong intuitive consensus against the utilitarian option is a common feature of many thought experiments that—unlike trolley dilemmas—were specifically devised by critics of utilitarianism in order to highlight utilitarianism’s counterintuitive implications. One such example—the “transplant” case, where one is asked whether to kill one person and use his organs to save five others—was actually included in the original battery of personal dilemmas. One might think that, in terms of its content, this dilemma is highly suitable for studying the contrast between utilitarian and deontological judgments. Yet, because almost no one thinks that such an act is morally acceptable, this dilemma is classified as low conflict, and thus excluded from studies that focus only on high conflict dilemmas.

In their interesting recent paper in this journal, Rosas and Koenigs (2014) highlight further problems with this stimuli set: even after the most problematic or irrelevant dilemmas have been removed, a significant number of high conflict dilemmas still fail to present a clean choice between a utilitarian act that maximizes aggregate welfare and a deontological option. This is because the supposedly utilitarian option in these dilemmas could also be supported by factors that are either irrelevant from a strict utilitarian perspective, or are even opposed to a utilitarian approach.

For example, Rosas and Koenigs point out that some high conflict dilemmas involve a strong component of self-interest: the sacrificial act saves not only the lives of strangers, but one’s own life. If subjects endorse this act, they needn’t be driven by the aim of maximizing the greater good; they might be just concerned about their own good. In other personal dilemmas, the person to be sacrificed would die anyway, so the choice is really between them dying and five others dying as well, or them dying and the five getting saved. This feature of these dilemmas is irrelevant from a simple utilitarian perspective, yet it may offer a strong independent reason to endorse the sacrificial act—a reason that some non-utilitarian ethicists endorse. Finally, in some dilemmas the person to be sacrificed is (directly or indirectly) the source of the threat to those who would be saved by the sacrificial act. Thus, in these dilemmas the person to be sacrificed is far from innocent, and may therefore lose the “moral immunity” normally possessed by an innocent bystander. This, again, is a moral factor that should be irrelevant from a straightforward utilitarian standpoint.

An immediate consequence of the above is that the supposedly “utilitarian” option in many high conflict dilemmas still fails to offer a clear contrast between utilitarian and deontological considerations, since the supposedly utilitarian choice can also be supported by strong self-interested reasons, by considerations (such as inevitability) that are also endorsed by many non-utilitarians, or even by explicitly non-utilitarian (i.e. deontological) moral considerations, relating to the guilt of a threatening agent. This therefore casts some doubt on the interpretation of prior studies reporting a supposed utilitarian bias in clinical populations of patients with damage to the ventromedial prefrontal lobe (Koenigs & Tranel, 2007) and of psychopaths (Koenigs et al., 2012). It would be surprising if psychopaths exhibit an unusually strong concern for the greater good; it is not that surprising that they exhibit an unusually strong concern for their own good (see also Kahane, 2014; Kahane, Everett, Earp, Farias, & Savulescu, 2015).

Rosas and Koenigs make a valuable contribution. But they do not go far enough. They want us to move “beyond utilitarianism,” and use the “impure” sacrificial dilemmas to study not utilitarian judgment but other distinctive patterns of response in clinical populations—I will consider this proposal at the end. But Rosas and Koenigs also give the impression that if researchers would just focus on those personal dilemmas that are “pure,” these dilemmas *could* be used to study utilitarian decision-making, or to identify a “utilitarian bias” in clinical populations. The problem they highlight is important, but it can be easily addressed by refining the dilemmas we use, weeding out the influence of irrelevant moral factors. Unfortunately however the problem with using sacrificial dilemmas to study utilitarian judgment goes far deeper. It cannot be addressed by any simple refinement of stimuli.

## DIGGING DEEPER: MISINTERPRETING ORDINARY MORAL THINKING

In the current literature, sacrificial dilemmas are almost invariably interpreted by reference to the contrast between philosophical theories such as the utilitarianism of Bentham and Mill and Kant’s deontology. But that such dilemmas can be used to highlight this contrast *in the philosophical context* does not automatically mean that this contrast is an illuminating way to interpret the responses of ordinary folk to such dilemmas. After all, utilitarianism and Kantian ethics are abstract theories that were first proposed a couple of hundred years ago in the West, and have never won the adherence of more than a tiny minority. It is doubtful, to say that least, that the forms of moral thinking that they recommend play much of a role in the moral thinking or ordinary people.

Philosophers sometimes contrast such ethical theories with what they call “commonsense morality”—the pre-theoretical moral views of the folk. Needless to say, commonsense morality is hardly a unity, let alone an abstract theory. But despite its messy diversity, it is characterized by a number of key features:

- Commonsense morality is clearly *not utilitarian*: it obviously does not have as its sole aim the maximization of the aggregate welfare of all sentient beings.
- Commonsense morality is *pluralist*: it recognizes a plurality of fairly specific moral rules and considerations, not a single abstract principle like Bentham’s Principle of Utility or Kant’s Categorical Imperative. (This pluralism is shared by some classical deontological theories, such as Ross, 1930/2002.)^4^ ^4^Since commonsense morality is not an explicit theory, the implicit rules that make it up need not be accessible to introspection or easy to articulate—just as our concepts of knowledge or causality clearly have a complex structure that is nevertheless difficult to spell out, even after centuries of philosophical reflection.
- Consequently, since these different moral rules sometimes conflict, commonsense morality does not always treat these rules as *absolutely* binding. In some contexts, one of these rules can outweigh or overrule another. Thus, while commonsense morality is deontological (in the loose sense of not being utilitarian), it is not based on a set of absolute prohibitions. For example, most people think that it is generally wrong to lie, but few believe that it is absolutely wrong to lie, in *all* circumstances. That rigid Kantian deontological view is as much a departure from commonsense as is the utilitarian view that we should always lie when this would lead to a better outcome (Kahane, 2012; Kahane et al., 2012). The current literature often identifies a deontological approach with such absolute prohibitions. This is a mistake.
- Commonsense morality (like many other deontological views) gives great moral significance to the prevention of harm and, more generally, to the promotion of people’s welfare. And it gives moral weight to numbers: saving more lives is morally better than saving less, helping many is better than helping few. This moral concern for others’ welfare has traditionally been referred to as the duty of beneficence, and is a feature even of Kantian ethics.^5^ ^5^Commonsense morality further distinguishes more stringent duties to prevent suffering and harm from weaker duties to confer benefits and promote happiness—a familiar moral distinction that utilitarians reject.
  This pedestrian moral idea has little to do with utilitarianism. It is not even correctly described as a utilitarian component *within* commonsense morality. What is distinctive of utilitarianism is not that it gives moral significance to welfare, not even that it gives weight to numbers. What is distinctive about utilitarianism is, first, that it is a *maximizing* view, requiring us to always act in the way that would lead to the greatest possible amount of aggregate welfare, and second, that it is a radically *impartial* view, requiring us to treat the welfare of *everyone* as of equal importance, regardless of whether they are near or far, our children and friends or absolute strangers, human or animal. This is why utilitarianism is sometimes described as generalized (or universal) benevolence.
  Needless to say, these are *not* features of commonsense morality. Commonsense morality is not a maximizing view: we can often fulfill our moral obligations by doing enough to help others, where enough is significantly less than the maximum possible. And commonsense morality is, in some respects, profoundly partial, allowing us to give significant priority to our own self-interest and to the welfare of those near and dear to us—to prefer, for example, our family, or compatriots, to distant strangers.

- According to commonsense morality it *is* sometimes permissible to overrule some deontological principle if following it would lead to great harm. This is especially true in emergency situations when the harm which would be prevented is very significant (think of medical triage). To illustrate, very few people would endorse Kant’s counterintuitive claim that it is wrong to lie even if this is necessary to prevent a murder.

In the current literature, when subjects judge that it is acceptable to sacrifice one person to save a greater number, this is classified as a utilitarian judgment, and thought to reflect a utilitarian cost–benefit analysis, which is argued by some to be uniquely based in deliberative processes (Cushman, Young, & Greene, 2010), and even in a distinctive neural subsystem (Greene, 2008; Greene et al., 2004).

This, I will now argue, is a misinterpretation of what underlies such “sacrificial” judgments. In fact, it should now by easy to see that such judgments can be better explained in terms of commonsense morality, without the slightest reference to utilitarianism:

### Rejecting a deontological rule is not yet a step in a utilitarian direction

It is typically assumed that when subjects endorse such a sacrificial act, they are *rejecting* a deontological rule against harming others in a direct and personal manner. But even if subjects making such judgments are really rejecting such a deontological rule, that in itself is not yet a move in a utilitarian direction.

There are very many possible deontological rules, and pretty much everyone rejects at least some, even many: liberals rejects such rules relating to purity or hierarchy, libertarians reject some such rules relating to distributive justice, socialists reject such rules relating to property rights, and so forth. What is distinctive of utilitarianism isn’t that it rejects one or some deontological constraints on the maximization of utility, but that it rejects *all* of them (Kahane & Shackel, 2010).

To reject a specific rule relating to harming others is perfectly compatible with endorsing extreme deontological rules in other contexts. And we have no reason to think that subjects who supposedly exhibit a “utilitarian bias” reject all (or even more) deontological rules—in fact there is evidence that there is no correlation between rejection of these rules in sacrificial dilemmas and rejecting them in other contexts, for example, relating to lying (Kahane et al., 2012).

It is therefore misleading to speak of a “utilitarian bias,” as if this expresses some general pro-utilitarian tendency. We should, at the very best, speak instead of a utilitarian bias *in the context of sacrificial dilemmas*, allowing that there may be no such a moral bias (or even a contrary tendency) in other contexts.

### Supposedly utilitarian judgments in sacrificial dilemmas lack the impartiality that is distinctive of a genuine utilitarian outlook

Utilitarians reject many conventional moral rules. But this rejection is certainly not the core of a utilitarian perspective. Its core is the *impartial maximization of the good of all*. The rejection of various deontological rules is just a consequence of that radical moral goal. In fact, the rejection of conventional moral rules is a feature utilitarianism shares with other views that may otherwise be diametrically opposed to it—such as *egoism*, which is likely to be the normative view that dominates the thinking of psychopaths (Kahane et al., 2015).

But do we have any reason whatsoever to think that subjects who tend to make supposedly “utilitarian” judgments in sacrificial dilemmas view morality in more impartial terms compared to others? Not really. It is not only psychopaths and vmPFC patients who are more willing to endorse a “utilitarian” act when it also involves an element of self-interest, but also ordinary folk (Moore et al., 2008). And rates of “utilitarian” judgments are strongly influenced by whether they involve sacrificing (or saving) foreigners versus compatriots (Swann, Gómez, Dovidio, Hart, & Jetten, 2010), or strangers versus family members (Petrinovich, O’Neill, & Jorgensen, 1993)—let alone animals versus humans (Petrinovich et al., 1993). In a recent study, we examined this issue more directly by investigating the relation between a tendency to “utilitarian” judgment in sacrificial dilemmas and a wide range of measures of impartial moral concern for the greater good in other contexts—for example, willingness to give some of one’s money to reduce the suffering of people in need in poor countries, rejection of the idea that the needs of one’s family or compatriots have moral priority over those of distant strangers, or generally identifying more with the whole of humanity. We consistently found either no relation or a negative relation between “utilitarian” judgment and such impartial concern for the greater good (Kahane et al., 2015). But it was anyway rather fanciful to suppose that, if psychopaths do exhibit a “utilitarian” tendency in sacrificial dilemmas, then they must also hold that we should give away much of our money to people in need in Africa if that would make the world a better place.

In other words, the judgments that are now routinely classified as “utilitarian” do not actually exhibit one of the key features that distinguishes a genuine utilitarian view from ordinary moral concern for others’ welfare.

### Subjects who make “utilitarian” judgments need not be rejecting the opposing deontological rule

To make things worse, it is doubtful that many of the subjects who make “utilitarian” judgments actually *reject* the deontological rule against direct and personal harm. The common assumption that subjects are rejecting this rule is based on the mistaken conflation of deontology with absolute prohibition: if you are willing to violate some prohibition, you are clearly not treating it as absolute. But as we saw, many of the rules of commonsense morality are *not* absolute. They can sometimes be outweighed by other moral considerations including, in the context of emergency situations, the harm that will be prevented if these rules are set aside.

That most people who make “utilitarian” judgments do not simply reject that deontological rule (as utilitarianism requires) is clearly shown by the fact that only very few of the participants of studies using sacrificial dilemmas make utilitarian judgments across the board—participants usually make a mix of utilitarian and deontological judgments, changing their mind from case to case. If these participants were simply rejecting a rule against direct and personal harm, such a pattern of response would make no sense (Kahane, 2012).

### Overruling a moral rule in emergency context when lives are at stake is part of commonsense morality

Commonsense morality offers no precise formula for deciding when a given moral rule is outweighed by another, and this can often be a matter of considerable disagreement—people will disagree, for example, on how much harm needs to be prevented for a white lie to be permissible. Most (but probably not all) ordinary folk would endorse pushing a man from a footbridge if that would save a thousand lives, or even dozens. Fewer people, it appears, endorse such acts in order to save only five lives. But, given what I’ve said above, it is doubtful that the latter judgments are qualitatively different from the former. They just involve a different understanding of what counts as preventing sufficient harm to justify overruling this moral rule in the context of such an emergency situation (Kahane, 2012).

### “Utilitarian” judgments in sacrificial dilemmas do not aim to maximize aggregate welfare

In the current literature, it is widely assumed that when subjects make “utilitarian” judgments, then this is the result of a utilitarian cost–benefit analysis. It is assumed, in other words, that because these subjects endorse the option that would lead to greater welfare (“five lives saved is greater than one life”), then they must be aiming to maximize welfare. It should be obvious by now, however, that this interpretation is not really licensed by the evidence. If subjects who make “utilitarian” judgment are really aiming to maximize utility, they should also judge that we ought to violently sacrifice one person to save *two* others, or sacrifice *fifty* to save *fifty one*—as a genuine utilitarian should judge. But it is very unlikely, to put it mildly, that these views are endorsed by more than a tiny handful. Moreover, while utilitarianism *requires* us to *always* maximize utility, most ordinary folks who make supposedly utilitarian judgments appear to merely hold that it is *acceptable* or *permissible* to sacrifice one to save five—a far weaker claim (see e.g. Lombrozo, 2009; Royzman, Landy, & Leeman, 2015).^6^ ^6^Studies such as Greene et al. (2001) that ask participants whether the sacrificial act is “acceptable” cannot distinguish between the utilitarian view that this act is required and the much weaker view that it is permissible both to commit this act and to refuse to do so (Kahane & Shackel, 2010). But this is a critical distinction. In fact, Royzman et al. (2015) found that a greater tendency to greater reflection was associated *only* with judgments of permissibility.


It is therefore a mistake to interpret “utilitarian” judgments as based in strict cost–benefit analysis. These judgments are not driven by any such radical maximizing aim, but by the far more mundane duty of beneficence mentioned above or, even more narrowly, by the unremarkable commonsensical idea that, when we are in an emergency situation and can easily save the lives of others, we have a (prima facie) duty to do so (something sometimes known as the “duty of rescue”).

### Deliberative processing is needed to weigh competing moral rules, not to perform a utilitarian cost–benefit analysis

This is not the only problem with claims about utilitarian cost–benefit analysis in this empirical literature. One influential strand of research not only ties “utilitarian” judgment to such cost–benefit analysis, but also claims that such analysis is uniquely tied to effortful deliberative processing—to be contrasted with the more primitive emotional responses that supposedly drive opposing deontological judgments (see for example, Greene, 2008; Greene et al., 2004).

It is rather odd however to think that it takes any kind of effortful cognition to calculate that five lives is greater than one life, or to think that only subjects who end up endorsing “utilitarian” conclusions make this trivial calculation. In fact, for a genuine utilitarian, sacrificial dilemmas should require no effort at all—*there is no dilemma*, and all one needs to do is to identify the course of action that would lead to most utility, an utterly straightforward decision in this context (Kahane, 2012).

If special cognitive effort is involved in arriving at such “sacrificial” moral conclusions, it is not likely to reflect the calculation of which option would lead to the better outcome (a trivial matter) but rather the weighing of several competing moral considerations—a particularly salient deontological principle telling us that we mustn’t cause certain kinds of harm (a component of what is often known as the duty of non-maleficence), and an opposing duty to prevent grievous harm to others (a component of beneficence)—actually a more complex form of moral deliberation than the mechanical application of utilitarian reasoning.^7^ ^7^An alternative explanation is that such effort is needed to overcome a persistent emotion or intuition that subjects take to be morally spurious; but see Kahane (2012) to see why this is an implausible explanation of the majority of cases. Notice that in a given context, some moral considerations or rules may be more salient than others, and thus have greater intuitive force, thereby either blocking further deliberation or dominating (or biasing) such deliberation. It does not follow from this, however, that the opposing moral considerations must therefore have a qualitatively different psychological character or source. It is this conflict of opposing duties or principles that makes these cases genuine dilemmas for most people—but neither of the moral rules involved has much to do with utilitarianism, a view that, as we just saw, *denies* that such situations involve any kind of genuine dilemma (Kahane, 2012).^8^ ^8^It is sometimes claimed that brain areas implicated in deliberative processing, such as the Dorsolateral Prefrontal Cortex (DLPFC), are involved in using “utilitarian” cost–benefit analysis to override, not only pre-potent “deontological” intuitions, but also pre-potent selfish impulses. But different kinds of responses will be pre-potent/intuitive in different contexts and populations. In some contexts, such as the Ultimatum Game, deliberative processing is needed to override a self-interested impulse and reject a beneficial yet unfair offer (Knoch, Pascual-Leone, Meyer, Treyer, & Fehr, 2006)—which is arguably a *deontological* response (Kahane & Shackel, 2010). And in some contexts, deliberative processing is needed to override pre-potent cooperative impulse, in order to arrive at a (counterintuitive) *selfish* decision (Rand, Greene, & Nowak, 2012; Suzuki, Niki, Fujisaki, & Akiyama, 2011). But such a general tie between deliberative processing and counterintuitive judgments is utterly unsurprising, and tells us nothing about utilitarian judgment *per se*; it just so happens that utilitarianism is associated with many counterintuitive moral conclusions. For further discussion, see Kahane et al. (2012), Kahane (2014).


## CONCLUSION: WHAT NEXT?

There is now a large and growing literature using sacrificial dilemmas to study utilitarian decision-making. The real problem with this literature is not that some of these dilemmas are problematic (although this is a serious issue), but that *sacrificial dilemmas tell us very little about utilitarian decision-making*. The mistake is to artificially project utilitarianism, a radical and demanding philosophical theory, onto the psychology of ordinary folk. This is not merely a pedantic complaint about terminology. As I have argued, the conceptual framework that currently dominates much research in this area is misleading, leading researchers to misinterpret what is really an everyday, *non*-utilitarian moral concern in terms of a simplistic—and largely irrelevant—opposition between utilitarian and deontological judgments. Ironically, the form of everyday deliberation that I have suggested really underlies sacrificial judgments is actually richer and more complex than the mechanical utilitarian cost–benefit analysis that is mistakenly projected onto it.^9^ ^9^A common criticism of sacrificial dilemmas is that they are unrealistic (Bauman, McGraw, Bartels, & Warren, 2014). This is not the worry I have been raising: lack of realism is both a disadvantage and an advantage (because, e.g., far-fetched examples allow us to better isolate distinct moral variables that would often be entangled in more realistic cases, or because they allow us to investigate judgments that do not merely reflect social convention). The issues I have been raising would remain in force even if we were to devise highly realistic instances of sacrificial dilemmas.


I do not want to give the wrong impression that past and current research on sacrificial dilemmas is completely misguided, or of no interest at all. I have argued that it tells us very little about utilitarianism (let alone offers any grounds for endorsing a utilitarian approach to ethics), but it certainly tells us something about the structure and psychological basis of certain commonsensical constraints on when it is morally permissible to harm others, and about when and why some people adhere to these constraints and others don’t. This is an interesting if rather narrow and unusual part of ordinary morality—it is not particularly central even to the domain of the ethics of harming, a vast and rich domain that ranges from questions about abortion and euthanasia to self-defense and collateral damage, and many other issues in between.

Moreover, the problematic conceptual framework that currently dominates research in this area obscures some important avenues of research. Instead of classifying judgments as utilitarian or deontological, and seeing these as based in utterly distinct neural subsystems or processes, we should try to investigate how different moral considerations are integrated and (when they are in conflict) weighed against each in moral deliberation. Do such moral rules have fixed, invariant weighs or is the weighing process more ad hoc and contextual? Which processes are involved in deciding that a given moral rule has been outweighed by another, and are they different from the processes that drive the outright rejection of a putative rule? Are there emotional processes that play a role—even an essential role—in everyday moral deliberation, for example, in resolving such moral conflicts? These are just some of the questions that could (and should) be investigated but that, so far as I can see, have been overlooked so far.

Sacrificial dilemmas are a peculiar place to start if one wants to investigate ordinary moral cognition. I have argued here that they are not even the right place to start if one wants to investigate utilitarian decision-making. Where should we start, then, if we want to investigate proto-utilitarian tendencies in everyday moral thinking? We should begin, I would suggest, with what is genuinely distinctive of utilitarian moral thinking. Not with the utilitarian’s willingness to dismiss conventional moral rules and norms—which, as we saw, is not only not the core of the view but is actually something utilitarianism that happens to share with very different views, meaning that research focusing on this dimension of utilitarianism risks ending up studying the psychology of views such as egoism, utilitarianism’s very opposite.

One of the things that *are* distinctive of utilitarianism is its radical impartiality—utilitarianism asks us to transcend our narrow focus on ourselves and those near and dear to us, and to extend our circle of concern to everyone, however geographically, temporally or even biologically distant. Strangely enough, however, this key aspect of utilitarianism has been virtually entirely ignored by current research on “utilitarian” judgment. The psychological basis of a radically impartial attitude to morality is, I believe, a fruitful area for future research. But it is doubtful that sacrificial dilemmas are a useful way to investigate this issue—and similarly doubtful that the psychological factors that dispose some individuals to adopt a more expansive view of morality are similar to those that drive supposedly utilitarian judgments in sacrificial dilemmas (Kahane et al., 2015).

Let me finally end by remarking on the Rosas and Koenigs (2014) suggestion that since many sacrificial dilemmas turn out not to cleanly pit utilitarian and deontological options due to the presence of interfering factors such as self-interest, inevitability of harm, or the guilt of the person to be sacrificed, we should move “beyond utilitarianism” and use these dilemmas to study the influence of these further factors on moral judgment in clinical (and presumably non-clinical) populations. Rosas and Koenigs (2014) provide suggestive evidence that vmPFC patients and psychopaths may exhibit a distinct pattern of moral judgment when these factors are present, a pattern that may be driven by abnormal affective responses. These preliminary results are certainly intriguing, and call for further research. It seems to me doubtful, however, that convoluted sacrificial dilemmas are the best way to investigate these issues.

Just to illustrate, consider the way considerations of self-interest might affect moral judgment in clinical or healthy populations. They might influence moral judgment covertly, in the form of a moral inconsistency: subjects may reject or endorse the very same moral conclusions depending on whether this is in their self-interest. Or self-interest may influence moral judgment *overtly* given that, as I explained above, commonsense morality sees certain forms of partiality as legitimate: we are often entitled to refuse to make great sacrifices even when this benefits others, and we are entitled to give priority to family, loved ones, and friends over mere strangers. But different people—and different subject populations—are likely to draw these lines in different places, disagreeing over when, for example, some self-sacrifice is too great, or justified partiality becomes mere nepotism. If considerations of self-interest influence the moral judgment of psychopaths to a greater degree than other populations, is this influence covert or also overt? If, compared to other populations, psychopaths give greater moral priority to their self-interest, might they, given their weaker ties to other people, also at the same time be *more* impartial when it comes to giving such priority to family and friends over strangers?^10^ ^10^For suggestive evidence supporting this hypothesis see Kahane et al. (2015). It is hard to see why, in investigating these and similar questions about moral egocentricity (and partiality more generally), we should rely on sacrificial dilemmas that were, after all, designed to address very different questions, and that involve self-interest (and the other factors highlighted by Rosas and Koenigs) only by oversight; we should not make the error of continuing to use this paradigm simply because it has dominated recent research.^11^ ^11^Similar points apply to investigating the way an agent’s guilt, or otherwise their being a threat to others, can make that agent less immune to harm. It seems plausible, however, that standard sacrificial dilemmas can be useful for studying the way the inevitability of harm affects judgments of permissible harming. We should move, not beyond utilitarianism, but beyond runaway trolleys

No potential conflict of interest was reported by the author.
